# Editorial: The Impact of Angiogenic Growth Factors and Hypoxia on the Antitumor Immune Response

**DOI:** 10.3389/fimmu.2022.903105

**Published:** 2022-04-19

**Authors:** Loredana Albonici, Sara Labiano Almiñana, Toshihide Tanaka, Roberto Bei

**Affiliations:** ^1^ Department of Clinical Sciences and Translational Medicine, University of Rome “Tor Vergata”, Rome, Italy; ^2^ Department of Pediatrics, Clinica Universidad de Navarra, Health Research Institute of Navarra (IDISNA), Pamplona, Spain; ^3^ Program in Solid Tumors, Foundation for the Applied Medical Research, Pamplona, Spain; ^4^ Department of Pediatrics, Clínica Universidad de Navarra, Pamplona, Spain; ^5^ Department of Neurosurgery, Jikei University School of Medicine Kashiwa Hospital, Kashiwa-shi, Japan

**Keywords:** angiogenic growth factors, hypoxia, TME (tumor microenvironment), anti-tumor innate immune cell, antitumor adaptive immune cells

Among the microenvironmental factors playing a role in cancer cells behavior, hypoxia is one of the most relevant. Hypoxia is a common feature of solid tumors triggered by an imbalance between cellular oxygen consumption and supply. Hypoxic tumor cells are able to trigger neoangiogenesis to meet the increased cellular oxygen demand. However, in addition to ensuring the growth and spread of cancer cells, neoangiogenesis results in aberrant and hyper-permeable vessels that reduce perfusion. Thus, the persistent high levels of angiogenic growth factors (AGFs), mainly VEGF, within the tumor microenvironment (TME) create a self-feeding circuit that supports a continuous hypoxic state thus allowing the generation of a hypoxic TME made by cellular stromal components, extracellular matrix (ECM) fibers, cytokines, and metabolic mediators, playing an important role in tumor development and progression. For these reasons, the impact of hypoxia target genes expression and function in the TME is becoming a complex area of research with the potential of unraveling novel promising hypoxic biomarkers as therapeutic targets.

Different therapeutic strategies targeting oxygen sensing and VEGF signaling, to restore both vessels normalization and antitumor immunity, are developed to improve the response to cancer immunotherapies. Immunotherapy is effective only in a fraction of patients and the combined therapeutic approaches have not always yielded the expected clinic results, leading to the need to identify new targets that could promote or hinder the antitumor immune response (Khouzam et al.)

In this regard, proteoglycans are ECM molecules capable of modulating the antitumor immune response. Syndecans (Sdcs), proteoglycans involved in the organization and assembly of ECM are dysregulated in cancer. Like VEGF, Sdc-3 production is promoted by the hypoxia inducible factor (HIF)-1α, it is mainly expressed by tumor-associated macrophages (TAMs) and is upregulated in solid tumor cells. Sdcs expression correlate with markers expressed by hot tumors such as IFN-γ and can predict a better patient overall survival (OS) in hypoxic tumors. Therefore, Sdc-3 might represent a useful marker of immunotherapy response in solid tumors (Prieto-Fernández et al.).

AGFs modulate the behavior of immune cells and, in turn, immune-mediated mechanisms regulate cells response to AGFs, making the two processes mutually interconnected. Therefore, AGFs produced in the hypoxic TME, create a tolerogenic setting that alters the normal hematopoiesis, thus favoring the expansion and recruitment of immature myeloid cells and promoting their functional exhaustion. In cancer patients, VEGF has been associated with the induction and maintenance of regulatory T (Treg) cells in TME, with a reduced differentiation and maturation of dendritic cells (DCs), and with the increase in myeloid-derived suppressor cells (MDSCs) in peripheral blood. These myeloid cells may be involved in further accumulation of Treg. Furthermore, hypoxic cells can evade immune attack by expressing specific molecules, such as immune checkpoint inhibitor (ICI) molecules, which block the activity of macrophages and effector T cells. As anti-angiogenic agents targeting VEGF signaling contribute to limit tumor-induced immunosuppression, different therapeutic strategies to restore an antitumor immunity arise from combined treatments of anti-angiogenic agents with immune checkpoint blockade leading to the improvement of these associations in different tumor types (Bourhis et al.).

The loss of HIF-1α, but not HIF-2α, reduces CD8^+^ T cells tumor infiltration and tumor cell killing. Nevertheless, the interaction between hypoxia, AGFs, and immune cells in TME is a complex scenario not yet fully understood. HIF-1α may function as a negative regulator of T-cell differentiation and cytokine production. However, the hypoxia-VEGF axis can also promote the presence of terminally exhausted CD8^+^ (exhCD8) T cells that under hypoxic conditions secrete VEGF, which in turn, increases the persistent expression of immune checkpoint inhibitory (ICIs) molecules, including PD-1, CTLA-4, and TIM-3. Interestingly, despite terminally exhCD8 T cells exhibit a highly cytotoxic-like phenotype they secrete less IFN-γ and IL-2 and are resistant to ICIs therapies. Therefore, targeting the mutual regulation between hypoxia, AGFs and immunosuppression can help to optimize the synergistic therapeutic combinations for improving the efficacy of anticancer treatments (Bannoud et al.).

Myeloid cells play a key role in the antitumor adaptive immune response due to their ability to modulate TME, in part *via* HIF transcription factors. As drugs targeting the HIF pathway are currently in clinical use, it is essential to understand the role played by hypoxia in the interrelationship between myeloid cells, adaptive immune cells, and TME, to better understand how they will affect patient outcomes. Strategies aimed at increasing the activation of the HIF pathway or pharmacological inhibitors of HIF-1α degradation in myeloid cells potently suppress T cell response and proliferation. Interestingly, HIF may have an anti-inflammatory role due to HIF-regulated myeloid (mainly macrophages) cell suppression of T cell-induced or -dependent tissue damage (Gojkovic et al.).

TAMs are the most abundant immune cells within TME. Macrophages migrate to accumulate in the most severely hypoxic regions of tumor tissue and adopt an M1-like pro-inflammatory phenotype early in oncogenesis by mediating an inflammatory immune response that inhibits tumor growth. As tumors progress, hypoxic TME gradually induces the M2-like functional polarization of TAMs that suppress Th1 immunity, have anti-inflammatory properties and contribute to tissue remodeling and angiogenesis. Among the many effects that hypoxic TME exhibits, there is the upregulation of TAMs PD-L1 expression, *via* HIF-1α, which promotes immunosuppression. Macrophages behavior is modulated by the cytokines type and levels present within TME in both normal and tumor tissues (He and Zhang).

In this regard, the cytokines family of interferons modulate both the antitumor immunity and the response to anticancer therapies in hypoxic TME. Type I IFNs, by promoting “immunogenic cell death”, can indirectly activate DCs, essential for CTLs activation. Besides, IFN-γ, by mediating “cancer immunoediting”, performs constant protective and sculpting actions of the immune response on developing tumors. However, if the IFN signaling persists, it acquires immunosuppressive features. Tumor cells require an active IFN signaling pathway for the success of many anticancer therapies and defects in IFN-γ signaling led to primary or acquired resistance to PD-1 and CTLA-4 blockage therapy (Arnaiz and Harris).

Overall, these novel Insights can help identify more effective cancer treatment strategies.

## Author Contributions

All authors listed have made a direct and intellectual contribution to the work and approved the submitted version.

## Conflict of Interest

The authors declare the absence of any commercial or financial relationships that could be construed as a potential conflict of interest.

## Publisher’s Note

All claims expressed in this article are solely those of the authors and do not necessarily represent those of their affiliated organizations, or those of the publisher, the editors and the reviewers. Any product that may be evaluated in this article, or claim that may be made by its manufacturer, is not guaranteed or endorsed by the publisher.

